# Rapid Eye Movement (REM)-Predominant Obstructive Sleep Apnea (OSA) Coexisting With and Initially Obscuring Narcolepsy Type 1: A Nine-Year Longitudinal Case Report

**DOI:** 10.7759/cureus.114706

**Published:** 2026-08-18

**Authors:** Abir Bouhamdi, Souad Aalil, Yassin Chefchaou, Sanae Labyad, Meryem Benjelloun, Abdellah Abida, Zouhayr Souirti, Mounia Serraj, Bouchra Amara, Mohamed Chakib Benjelloun, David Gozal, Mohamed ElBiaze

**Affiliations:** 1 Department of Pulmonology, Hassan II University Hospital, Sidi Mohamed Ben Abdellah University, Fez, MAR; 2 Department of Pulmonology, Hassan II University Hospital, Fez, MAR; 3 Department of Neurology, Hassan II University Hospital, Fez, MAR; 4 Department of Pediatrics, Marshall University, Joan C. Edwards School of Medicine, Huntington, USA; 5 Department of Pneumology, Hassan II University Hospital, Fez, MAR

**Keywords:** cataplexy, excessive daytime sleepiness, multiple sleep latency test, narcolepsy type 1, obstructive sleep apnea, rem-predominant obstructive sleep apnea

## Abstract

Obstructive sleep apnea (OSA) is a common disorder whose clinical expression and daytime sleepiness may evolve over time, particularly in the presence of coexisting sleep disorders. We report a nine-year longitudinal case of a 29-year-old man presenting initially with severe excessive daytime sleepiness (EDS) (Epworth Sleepiness Scale (ESS) score: 19/24) and mild OSA (apnea-hypopnea index (AHI): 10 events/h), who represented nine years later with disabling sleepiness, weight gain (body mass index: 30.2 kg/m²), and typical cataplexy. No documented sleep medicine follow-up or OSA treatment was available during the intervening nine-year period.

Repeat polysomnography demonstrated moderate, rapid eye movement (REM)-predominant OSA (AHI: 20.4 events/h; REM-AHI: 52.9 events/h; non-REM (NREM)-AHI: 9.4 events/h; REM-AHI/NREM-AHI ratio: 5.6), with increased sleep fragmentation. The Multiple Sleep Latency Test (MSLT), performed after diagnostic polysomnography and before continuous positive airway pressure (CPAP) optimization, showed severe objective sleepiness (mean sleep latency: 3.7 minutes) with sleep-onset REM periods in all five nap opportunities. In the setting of typical cataplexy, these findings strongly supported a diagnosis of narcolepsy type 1; however, untreated OSA and sleep fragmentation at the time of testing were acknowledged as potential confounders in the interpretation of the MSLT.

Management included fixed-pressure CPAP at 8 cm H₂O, sertraline for cataplexy, and modafinil for EDS. At 30-day follow-up, CPAP downloads showed use on 60% of nights, with a residual AHI of 3.1 events/h and mean leak of 35 L/min, indicating effective control of respiratory events when therapy was used, but suboptimal adherence and mask leak likely limiting clinical benefit.

This case highlights three key clinical messages: EDS disproportionate to OSA severity should prompt evaluation for a coexisting central disorder of hypersomnolence; REM-predominant OSA can coexist with narcolepsy type 1 without implying a causal relationship between the two disorders; and their relative contributions to sleepiness may change over time. When MSLT is required in a patient with OSA, effective treatment of OSA and adequate documentation of prior sleep are important for reliable interpretation. Management should address both OSA and narcolepsy and include periodic reassessment as the clinical phenotype evolves.

## Introduction

Obstructive sleep apnea (OSA) is a common and complex disorder characterized by recurrent upper airway obstruction during sleep, leading to sleep fragmentation and intermittent hypoxemia. Its global burden is substantial, with nearly one billion individuals estimated to be affected [1]. Beyond impaired quality of life and excessive daytime sleepiness (EDS), OSA is associated with cardiometabolic and neurobehavioral sequelae, as well as increased motor vehicle and occupational accident risk. Importantly, the relationship between subjective sleepiness, assessed using the Epworth Sleepiness Scale (ESS), and OSA severity, measured by the apnea-hypopnea index (AHI), is modest; prominent EDS may occur despite low-to-moderate AHI values [2-4].

Narcolepsy type 1 is characterized by severe EDS, rapid eye movement (REM) sleep dysregulation, and cataplexy, and is strongly associated with loss of hypothalamic hypocretin (orexin) neurons [5]. Because OSA is highly prevalent, the two disorders may coexist. In a retrospective study of adults with narcolepsy and OSA, REM-related OSA was common [6]; however, this association does not establish a causal relationship between narcolepsy and REM-predominant OSA.

Experimental animal data suggest that orexin signaling may contribute to upper airway motor control. In rat models, orexin A activates hypoglossal motoneurons and enhances genioglossus muscle activity [7]. These experimental findings provide biological plausibility for a potential relationship between orexin deficiency and upper airway vulnerability; however, this mechanism has not been established in humans and should not be interpreted as a proven causal pathway linking narcolepsy to REM-predominant OSA [6,7].

Here, we report the longitudinal course of a 29-year-old man initially presenting with severe EDS and mild OSA, who represented nine years later with disabling EDS, typical cataplexy, and moderate REM-predominant OSA. This case highlights changes in the clinical and polysomnographic phenotype over time and underscores the importance of recognizing coexisting sleep disorders. It also emphasizes the need to evaluate for narcolepsy when EDS appears disproportionate to AHI and to adopt a multimodal approach that optimizes OSA treatment while addressing central hypersomnolence.

## Case presentation

Mr JA, a 29-year-old banker, was first referred to the Sleep Medicine Center at Hassan II University Hospital, Fez, Morocco, in 2016 by a neurologist for persistent EDS. At that time, he reported an approximately seven-year history of hypersomnolence without cataplexy. He had no known medical comorbidities. Neurological investigations, including brain magnetic resonance imaging and electroencephalography, were unremarkable.

Initial otorhinolaryngologic examination showed normal facial morphology, Class I dental occlusion, Mallampati Class II, Friedman stage II, patent nasal passages, and no visible velopharyngeal or retrolingual obstruction. Cephalometric assessment reported values within reference ranges (MP-H 19.3 mm; Ba-S-N 133.7°; ANB 5.1°; PAS 10.8 mm; SNA 83°; SNB 77.9°).

Initial nocturnal polysomnography demonstrated mild OSA, with an AHI of 10 events/h and a respiratory disturbance index (RDI) of 14.4 events/h. Subjective sleepiness was disproportionately high, with an ESS score of 19/24, Pichot fatigue scale score of 13/32, and Patient Health Questionnaire-9 (PHQ-9) score of 5/27, despite only mild OSA. This discrepancy prompted consideration of a central disorder of hypersomnolence, including narcolepsy without cataplexy versus idiopathic hypersomnia. A sleep diary and 24-hour polysomnography were advised; however, the patient was lost to follow-up.

During the subsequent nine-year interval, the patient did not seek further medical evaluation for his sleep-related symptoms and received no treatment for OSA. He later reported that EDS had persisted throughout this period and had worsened more recently. The precise timing of cataplexy onset could not be reliably established retrospectively.

Nine years later, in 2025, he returned because of worsening and disabling daytime sleepiness, including unintended sleep episodes at work and while driving. His symptoms had begun to interfere substantially with his occupation as a banker, requiring repeated checks of financial documents because he was concerned that impaired vigilance could lead to a serious professional error. He also reported dream-enriched naps and typical cataplexy, characterized by brief episodes of bilateral knee buckling and head dropping triggered by strong emotions such as laughter and without loss of consciousness. Loud nocturnal snoring was also reported. Additional symptoms included irritability, mood fluctuation, reduced concentration, and memory complaints. There were no nightmares, sleep paralysis, or hypnagogic or hypnopompic hallucinations.

At representation, his weight was 98 kg and height was 1.80 m, corresponding to a body mass index of 30.2 kg/m², compared with 24.0 kg/m² at the initial assessment. Blood pressure was 120/80 mmHg in both arms, heart rate was 71 beats/min, and resting oxygen saturation was 96% on room air. Electrocardiography and transthoracic echocardiography were unremarkable. Updated otorhinolaryngologic examination showed normal facial morphology, Class I dental occlusion, Mallampati Class II, Friedman stage II, patent nasal passages without septal deviation or nasal polyps, and no visible retropalatal or retroglossal obstruction. Sleepiness and fatigue scores had worsened, with an ESS score of 22/24, Pichot fatigue scale score of 19/32, and PHQ-9 score of 11/27.

Repeat polysomnography demonstrated moderate OSA, predominantly during REM sleep, with reduced sleep efficiency and increased sleep fragmentation compared to the initial study. Total sleep time was 411.5 minutes, sleep efficiency was 76.1%, REM sleep accounted for 97.5 minutes (23.7% of total sleep time), and the arousal index was 58.2 events/h. For this report, REM-predominant OSA was operationally defined as an overall AHI ≥5 events/h, a REM-AHI/NREM-AHI ratio ≥2, and an NREM-AHI <15 events/h [6]. In this patient, the overall AHI was 20.4 events/h, REM-AHI was 52.9 events/h, NREM-AHI was 9.4 events/h, and the REM-AHI/NREM-AHI ratio was 5.6, supporting a REM-predominant pattern. The RDI was 50.9 events/h, with a respiratory effort-related arousal (RERA) index of 30.5 events/h. Oxygen desaturation was limited overall, with an oxygen desaturation index of 2.4 events/h, 0.2% of total sleep time spent below 90% oxygen saturation, and an oxygen saturation nadir of 82%.

**Figure 1:**
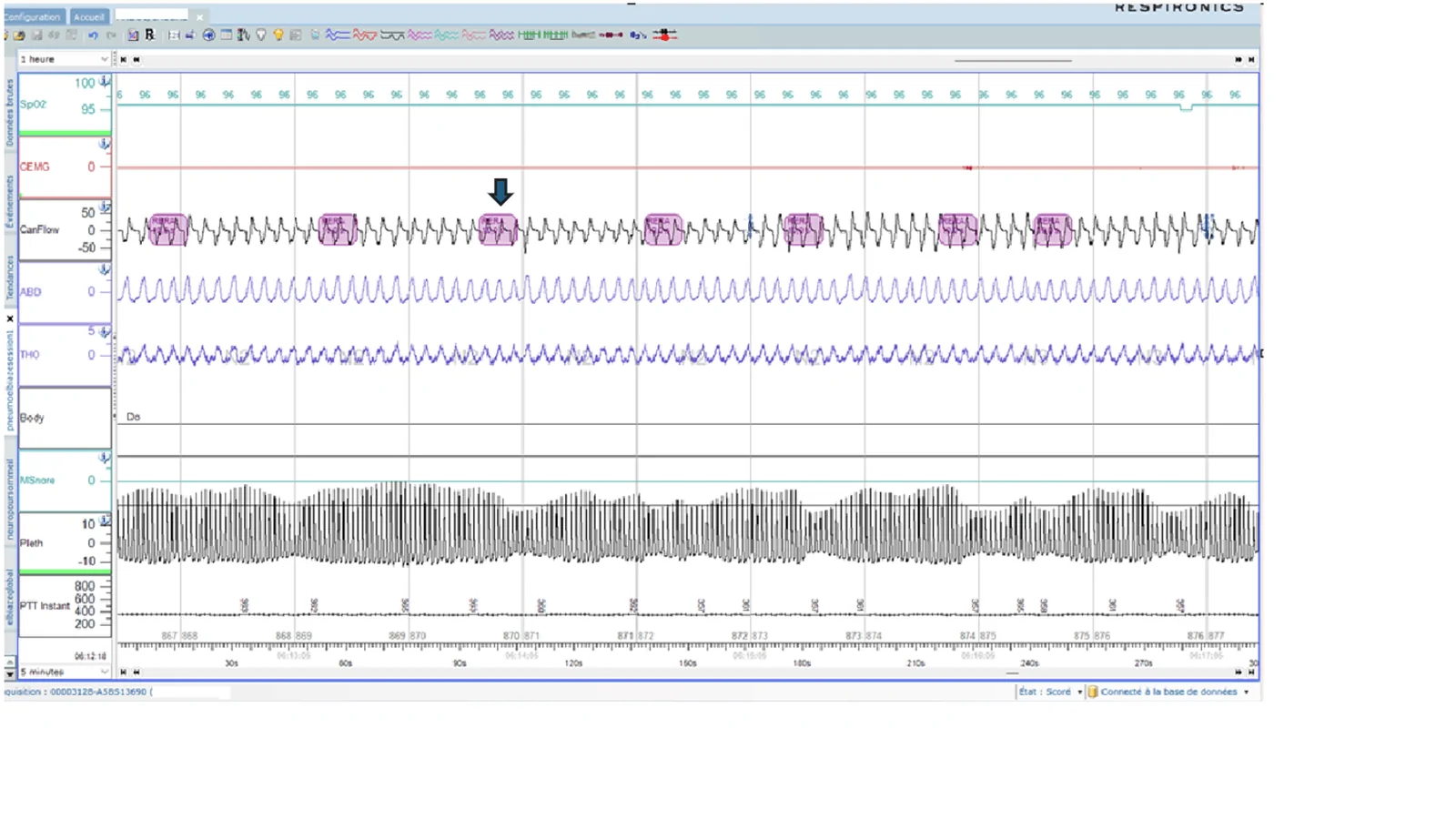
Representative polysomnographic tracing during REM sleep (a) Flow limitation preceding a respiratory effort-related arousal (RERA; boxed areas) REM: rapid eye movement; RERA: respiratory effort-related arousal.

**Figure 2:**
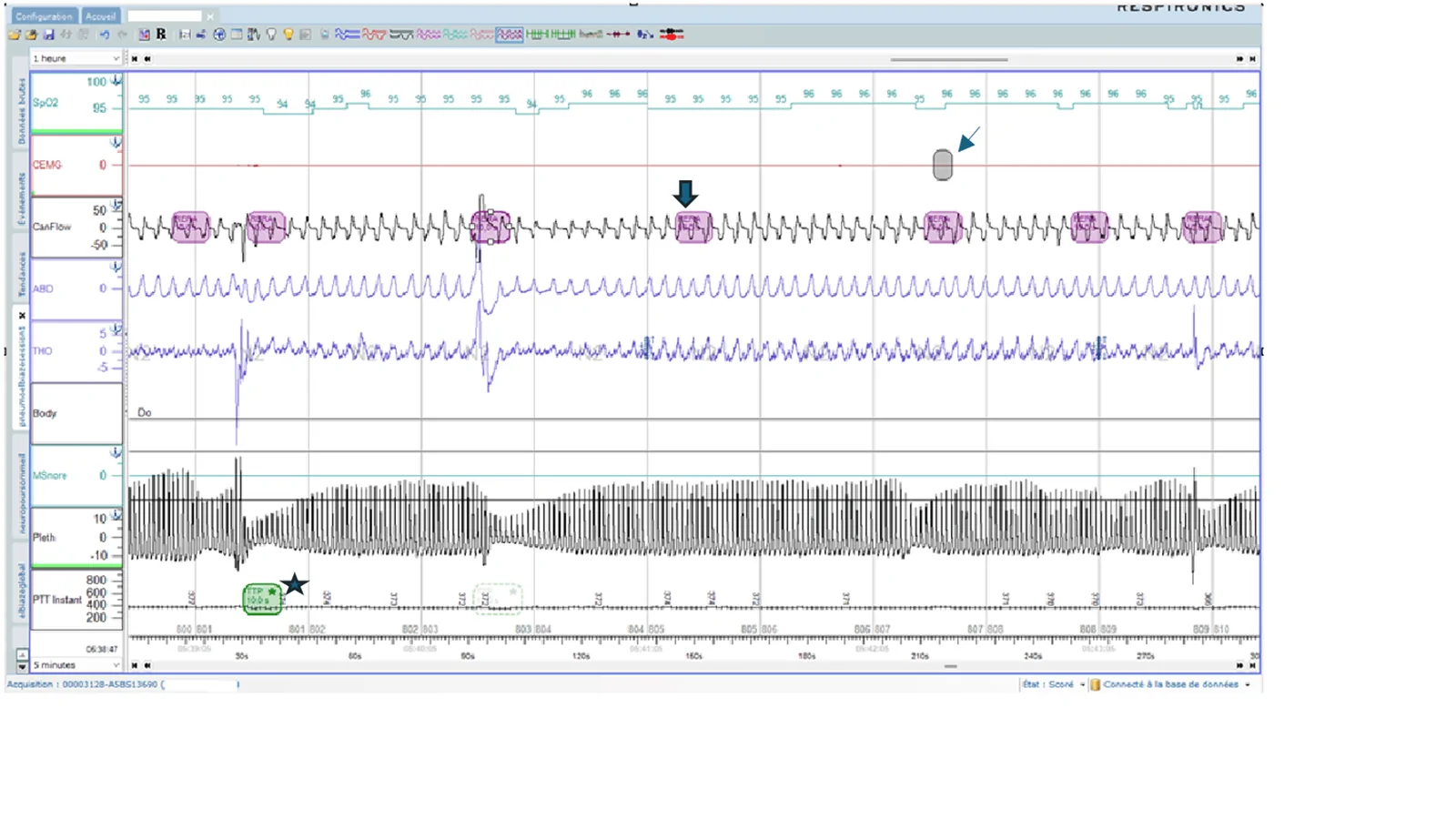
Representative polysomnographic tracings during REM sleep (b) RERA associated with cortical arousal (arrow) REM: rapid eye movement; RERA: respiratory effort-related arousal.

**Figure 3:**
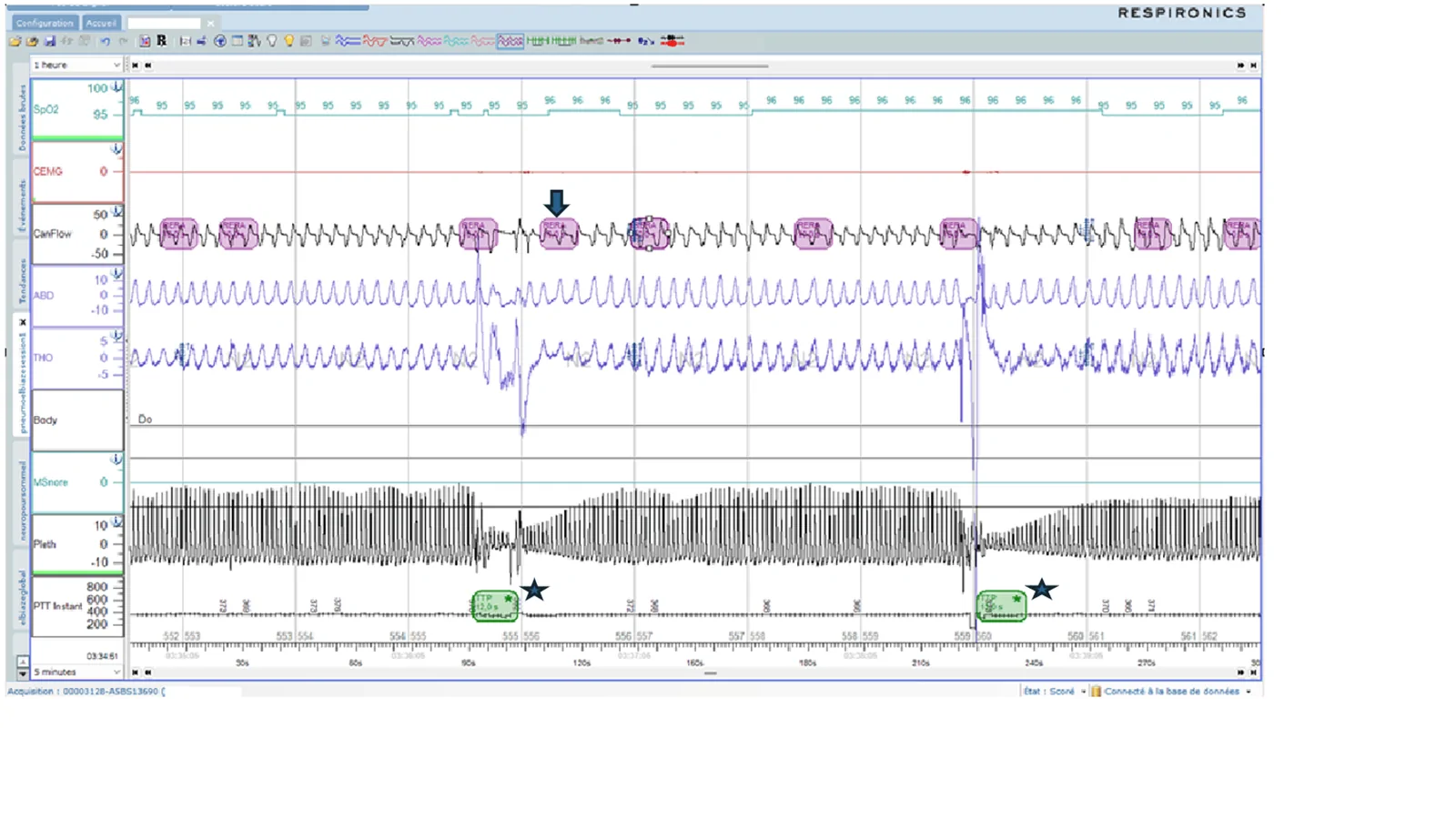
Representative polysomnographic tracings during REM sleep (c) RERA associated with a transient pulse transit time change (asterisk), reflecting autonomic activation REM: rapid eye movement; RERA: respiratory effort-related arousal.

The patient spent 271.4 minutes of sleep in the supine position and 140.1 minutes in non-supine positions. The supine AHI was 21.1 events/h, while the time-weighted non-supine AHI was approximately 15.5 events/h. Position-specific AHIs were 30.7 events/h during left lateral sleep, 12.1 events/h in the prone position, and 14.3 events/h during right lateral sleep, with corresponding sleep durations of 15.0, 32.8, and 92.3 minutes, respectively. Comparative polysomnographic parameters from the 2016 and 2025 studies are summarized in Table 1, and representative polysomnographic tracings during REM sleep are shown in Figures 1-3.

**Table 1:** Polysomnographic parameters at baseline and nine-year follow-up AHI: apnea-hypopnea index; NREM: non-rapid eye movement; ODI: oxygen desaturation index; PSG: polysomnography; RDI: respiratory disturbance index; RERA: respiratory effort-related arousal; REM: rapid eye movement; SpO₂: oxygen saturation; T90: percentage of total sleep time with oxygen saturation below 90%; TST: total sleep time.

Polysomnographic parameter	Initial PSG (2016)	Nine-year follow-up PSG (2025)
TST (min)	512.0	411.5
Sleep efficiency (%)	92.2	76.1
Sleep onset latency (min)	0	19.5
REM latency from sleep onset (min)	56	58.5
REM sleep (% TST)	11.4	23.7
Total arousal index (events/h)	25.1	58.2
Number of awakenings	35	25
Number of stage transitions	136	87
AHI (events/h)	10	20.4
Obstructive apnea index (events/h)	1.1	0.9
Hypopnea index (events/h)	8.8	18.7
REM-AHI (events/h)	12.3	52.9
NREM-AHI (events/h)	9.7	9.4
REM-AHI/NREM-AHI ratio	1.3	5.6
Supine AHI (events/h)	19.3	21.1
Non-supine AHI (events/h)	5.7	15.5
Supine sleep time (min)	161.8	271.4
Non-supine sleep time (min)	350.2	140.1
RDI (events/h)	14.4 (RERA: -4.4)	50.9 (RERA: -30.5)
Snoring duration (% of TST)	0.1	-
Mean sleep SaO₂ (%)	97	95
Oxygen saturation nadir (%)	84	82
T90 (% of TST <90% SpO_2_)	0	0.2
ODI 3% (events/h)	0.4	2.4

**Figure 4:**
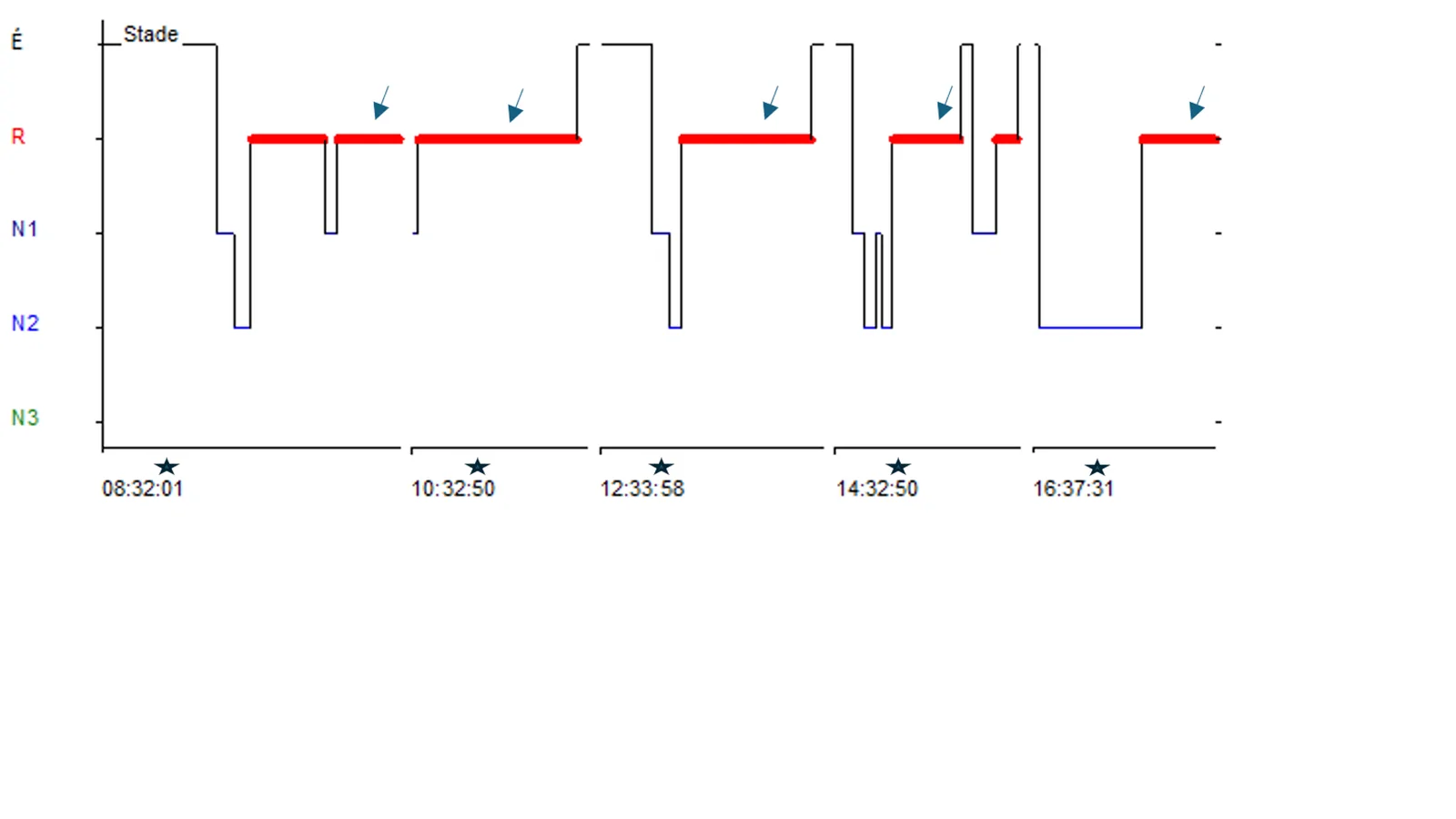
Multiple Sleep Latency Test hypnogram of the patient The x-axis represents time, and the y-axis represents sleep stages. Sleep-onset REM periods (SOREMPs) occurred in all five nap opportunities. Arrows indicate SOREMPs, and stars indicate the five nap opportunities. REM: rapid eye movement.

The Multiple Sleep Latency Test (MSLT) was performed the day after the diagnostic polysomnography during the same hospitalization and before continuous positive airway pressure (CPAP) optimization. The preceding polysomnography documented 411.5 minutes of total sleep time. A sleep diary completed during the weeks preceding polysomnography documented a regular sleep-wake schedule. The patient worked regular daytime hours as a banker, without shift work or clinical evidence of circadian misalignment. Before testing, he reported no caffeine, alcohol, cannabis, or other substance use and was not receiving medications, antidepressants, or other REM-modulating agents. No actigraphy was performed. Nevertheless, untreated OSA and substantial sleep fragmentation were considered potential confounders in the interpretation of sleep latency and sleep-onset REM periods (SOREMPs). The MSLT demonstrated severe objective sleepiness, with a mean sleep latency of 3 minutes 42 seconds and SOREMPs in all five nap opportunities. In the context of typical cataplexy and chronic disabling EDS, these findings strongly supported a diagnosis of narcolepsy type 1, while acknowledging the limitations related to untreated OSA and sleep fragmentation at the time of testing (Figure 4).

Alternative explanations for the patient's symptoms and MSLT findings were considered. Untreated OSA and sleep fragmentation may have contributed to EDS and to the MSLT abnormalities but did not fully account for the presence of typical emotion-triggered cataplexy. The preceding sleep diary and regular daytime work schedule did not suggest chronic insufficient sleep or a major circadian rhythm disorder. Depressive symptoms were considered potentially contributory but insufficient to explain the overall clinical phenotype. Brain magnetic resonance imaging and electroencephalogram were unremarkable. CSF hypocretin-1 measurement and HLA testing were not performed.

Treatment was initiated with fixed-pressure CPAP at 8 cm H₂O. Sertraline 50 mg/day was prescribed for cataplexy, and modafinil 200 mg/day was prescribed for EDS in the context of disabling occupational impairment and driving safety concerns. Sodium oxybate was not selected as initial therapy, while OSA treatment was being established and optimized.

At 30-day follow-up, CPAP device downloads showed use on 60% of nights, with a residual AHI of 3.1 events/h and a mean unintentional leak of 35 L/min. These findings indicated effective control of respiratory events when CPAP was used, although suboptimal adherence and elevated leak likely limited its overall clinical benefit. Targeted interventions, including mask refitting, leak correction, comfort optimization, and adherence coaching, were implemented. No standardized follow-up ESS score was recorded. The patient was subsequently lost to follow-up, precluding assessment of longer-term clinical response, treatment adherence, and evolution of daytime sleepiness and cataplexy.

Written informed consent was obtained from the patient for publication of the clinical details, polysomnographic data, MSLT hypnogram, and related images included in this case report.

## Discussion

This case illustrates the diagnostic and therapeutic complexity of coexisting OSA and narcolepsy type 1 and documents substantial changes in the clinical and polysomnographic phenotype over a nine-year interval. The patient progressed from mild OSA without REM predominance to moderate REM-predominant OSA, while body mass index increased from 24.0 to 30.2 kg/m² and daytime sleepiness became increasingly disabling. Longitudinal studies have shown that OSA clinical phenotypes may change over time and that changes in body weight are associated with changes in sleep-disordered breathing severity [8,9]. However, the temporal coexistence of weight gain, worsening OSA, and emergence of a REM-predominant pattern in this patient does not establish that one caused the other, nor can a single case determine whether narcolepsy contributed to the change in OSA phenotype.

Narcolepsy type 1 is strongly associated with loss of hypothalamic hypocretin (orexin) neurons and consequent instability of wakefulness and REM sleep [5]. REM-related OSA has been reported frequently among adults with narcolepsy and coexisting OSA [6]. A potential mechanistic relationship is biologically plausible but remains unproven. Experimental studies in rats have shown that orexin A can activate hypoglossal motoneurons and enhance genioglossus muscle activity [7]. These findings suggest a possible role for orexin signaling in upper-airway motor control, but they should not be extrapolated as evidence that hypocretin deficiency causes REM-predominant OSA in humans. Thus, the REM-predominant pattern observed in our patient should be interpreted as an associated phenotype rather than evidence of a causal pathway linking narcolepsy and OSA.

The marked difference between the AHI and RDI at the nine-year evaluation (20.4 versus 50.9 events/h) was largely attributable to a RERA index of 30.5 events/h. This finding indicates a substantial burden of arousal-associated respiratory events under the scoring approach used in our laboratory. However, it should not be interpreted as definitive evidence of a longitudinal increase in upper-airway resistance or arousal instability. Arousal-based respiratory scoring, RERA identification, and RDI definitions may vary according to scoring methodology, and different approaches can produce substantially different estimates of sleep-disordered breathing severity [10,11]. Accordingly, the increase in RDI in this patient should be interpreted cautiously and in conjunction with other polysomnographic findings, rather than as an independent marker of physiological progression.

The interpretation of the MSLT warrants particular caution. American Academy of Sleep Medicine (AASM) guidance recommends documenting adequate habitual sleep before testing and, in patients undergoing MSLT for persistent sleepiness despite OSA, ensuring that OSA treatment is established and effective [12]. In our patient, the MSLT was performed after a nocturnal polysomnogram documenting 411.5 minutes of sleep, a preceding sleep diary indicated a regular sleep-wake schedule, and there was no history of shift work, relevant substance use, or medications likely to suppress or alter REM sleep. Nevertheless, the MSLT was performed before CPAP optimization, in the presence of untreated moderate OSA and substantial sleep fragmentation. These factors may shorten sleep latency and potentially influence SOREMP occurrence and therefore represent important limitations of the test interpretation [12].

Despite this limitation, several features strongly supported narcolepsy type 1 in the overall clinical context: longstanding disabling EDS, typical emotion-triggered cataplexy, a mean sleep latency of 3 minutes 42 seconds, and SOREMPs in all five nap opportunities. Alternative explanations were considered. The preceding sleep diary and regular daytime work schedule did not suggest chronic insufficient sleep or a major circadian rhythm disorder; no relevant medication or substance exposure was identified before testing; and the mild-to-moderate depressive symptoms were considered potentially contributory to sleepiness but insufficient to explain typical cataplexy. Untreated OSA remained an important contributor to sleepiness and a methodological confounder for the MSLT. CSF hypocretin-1 measurement and HLA testing were not performed, and therefore no additional biomarker confirmation was available.

The coexistence of OSA and narcolepsy may also complicate interpretation of nocturnal sleep fragmentation. Narcolepsy itself is associated with disrupted nighttime sleep, spontaneous awakenings, and instability of sleep-stage transitions [13], whereas OSA produces respiratory-event-related arousals and sleep fragmentation. In an individual with both disorders, the relative contribution of each condition to nocturnal sleep disruption and daytime symptoms cannot be precisely determined from a single polysomnographic assessment. The substantial arousal burden in our patient should therefore be viewed as potentially multifactorial rather than attributed exclusively to either OSA or narcolepsy.

Management of coexisting OSA and narcolepsy requires treatment of both conditions. CPAP efficacy, adherence, mask leak, and pressure requirements should be optimized, while weight management remains relevant because longitudinal data demonstrate an association between weight change and OSA severity [9]. At the same time, clinically significant narcolepsy symptoms may require specific therapy rather than waiting for complete resolution of OSA-related sleepiness. Current AASM recommendations strongly support modafinil, pitolisant, sodium oxybate, and solriamfetol for the treatment of narcolepsy in adults [14]. European guidelines similarly strongly recommend modafinil for EDS and sodium oxybate for both EDS and cataplexy, while venlafaxine and clomipramine are also strongly recommended for cataplexy [15]. Sertraline, as used in our patient, represents an off-label anticataplectic approach with a less robust evidence base than these guideline-supported therapies.

Sodium oxybate was not selected as initial therapy in this patient, while treatment of his coexisting OSA was being established and optimized. Although sodium oxybate is an evidence-based treatment for both EDS and cataplexy [14,15], caution is appropriate in patients with sleep-disordered breathing. In a randomized safety study involving patients with mild-to-moderate OSA, sodium oxybate did not significantly worsen mean AHI or mean oxygen saturation overall, but an increase in central apneas and clinically significant oxygen desaturation occurred in some individuals [16]. In the setting of untreated OSA at the time of therapeutic decision-making, establishing effective CPAP before considering sodium oxybate was therefore a cautious individualized approach. Modafinil was selected to address disabling daytime sleepiness and immediate occupational and driving safety concerns, while sertraline was used for cataplexy.

Non-pharmacological management remains important and includes maintaining a regular sleep schedule, scheduled naps, weight management, and explicit counseling regarding driving and occupational safety [15]. Periodic reassessment is particularly important when OSA and narcolepsy coexist because the relative contribution of each disorder to symptom burden may change over time.

Several limitations should be acknowledged. First, this is a single case and cannot establish causal relationships among narcolepsy, REM-predominant OSA, or weight gain. Second, the nine-year loss to follow-up prevents precise reconstruction of the timing of cataplexy onset, the trajectory of weight gain, and the temporal relationship between these changes and worsening sleepiness, although the patient reported no interim sleep-medicine evaluation or OSA treatment. Third, although an adequate preceding polysomnographic sleep duration and a regular sleep schedule documented by sleep diary supported the MSLT assessment, the test was performed before OSA treatment was optimized and without actigraphic confirmation, limiting interpretation. Fourth, CSF hypocretin-1 and HLA testing were not performed. Fifth, RERA-based RDI measurements are dependent on scoring methodology and should not be overinterpreted longitudinally. Finally, CPAP follow-up was limited to 30 days, no standardized follow-up ESS score was obtained, and the patient was subsequently lost to follow-up, precluding assessment of longer-term treatment adherence, symptom response, and evolution of daytime sleepiness and cataplexy.

## Conclusions

This case highlights the diagnostic complexity of coexisting OSA and narcolepsy type 1. Severe EDS disproportionate to OSA severity should prompt evaluation for a central disorder of hypersomnolence, particularly when typical cataplexy is present. In patients with coexisting OSA, MSLT findings should be interpreted cautiously when OSA treatment has not yet been optimized, as untreated OSA and sleep fragmentation may confound the results. Management requires optimization of OSA therapy, targeted treatment of narcolepsy-related symptoms, and longitudinal reassessment. Changes in OSA phenotype, weight, and narcolepsy symptoms should be interpreted as temporal associations rather than causal relationships in a single case.
